# Identification of genome-wide binding sites of heat shock factor 1, Hsf1, under basal conditions in the human pathogenic yeast, *Candida albicans*

**DOI:** 10.1186/s13568-018-0647-7

**Published:** 2018-07-16

**Authors:** Remya Nair, Nitesh K. Khandelwal, Md. Shariq, Archana K. Redhu, Naseem A. Gaur, Shamim Shaikh, Rajendra Prasad

**Affiliations:** 1 Rajiv Gandhi Institute of IT & Biotechnology, Bharati Vidyapeeth University, Pune, 411045 India; 2 School of Life Sciences, Jawaharlal Nehru University, New Delhi, 110067 India; 3 International Centre for Genetic Engineering and Biotechnology, New Delhi, 110067 India; 4 National Institute of Pathology, Safdarjung Campus, New Delhi, 110029 India; 5 Department of Biosciences and Bioengineering, IIT-Bombay, Mumbai, 400076 India; 6 Amity Institute of Integrative Sciences and Health and Amity Institute of Biotechnology Amity University, Gurgaon, 122413 India

**Keywords:** Hsf1 (Heat shock factor 1), Iron, Drug resistance, Heat shock, Chaperones, Motif and *Candida albicans*

## Abstract

The master regulator of thermal stress response, Hsf1, is also an essential determinant for viability and virulence in *Candida albicans*. Our recent studies highlighted that apart from ubiquitous roles of Hsf1 at higher temperatures, it also has myriad non-heat shock responsive roles essential under iron deprivation and drug defense. Here, we further explored its implications in the normal cellular functioning, by profiling its genome-wide occupancy using chromatin immuno-precipitation coupled to high-density tiling arrays under basal and iron deprived conditions. Hsf1 recruitment profiles revealed that it binds to promoters of 660 genes of varied functions, under both the conditions, however, elicited variability in intensity of binding. For instance, Hsf1 binding was observed on several genes of oxidative and osmotic stress response, cell wall integrity, iron homeostasis, mitochondrial, hyphal and multidrug transporters. Additionally, the present study divulged a novel motif under basal conditions comprising, -GTGn_3_GTGn_3_GTG- where, Hsf1 displays strong occupancy at significant number of sites on several promoters distinct from the heat induced motif. Hence, by binding to and regulating major chaperones, stress responsive genes and drug resistance regulators, Hsf1 is imperative in regulating various cellular machineries. The current study provides a framework for understanding novel aspects of how Hsf1 coordinates diverse cellular functions.

**Electronic supplementary material:**

The online version of this article (10.1186/s13568-018-0647-7) contains supplementary material, which is available to authorized users.

## Introduction

Survival of a pathogen within the host relies on its ability to proficiently respond to various environmental stimuli it encounters. Microorganisms are perennially challenged with varied forms of stresses spanning from oxidative, osmotic, thermal, pH stress to nutrient limitation (Lafayette et al. 2010). Sensing temperature fluctuations and initiating the appropriate response is known to be a critical attribute of virulence in several pathogens (Leach and Cowen 2013). Response to heat shock is one of the fundamentally vital processes that has been highly conserved from yeasts to humans (Nicholls et al. 2011). On exposure to sudden thermal transitions, the cells are programmed to promptly induce the expression of molecular chaperones and other classes of proteins, which work in coordination to protect the cell from cellular damage, induced by heat shock (Leach et al. 2012). In eukaryotes, the evolutionarily conserved heat shock transcription factor 1 (Hsf1), a client protein of Hsp90, is the master regulator of thermal stress response and is established to activate heat shock proteins (HSPs) via canonical heat shock elements (HSEs) in their promoters.

Heat shock response in the human pathogen, *Candida albicans* is controlled mainly by the essential transcription factor, Hsf1 in co-ordination with the heat shock induced chaperones, chiefly Hsp90. Hsf1 in concert with Hsp90 has been recently established to modulate global transcriptional responses to stress via alterations in nucleosome architecture (Leach et al. 2016). *C. albicans* although is obligately associated with warm-blooded animals, has still retained a heat shock response and hence, may be presumed to have some other functions under basal conditions as well. The underpinnings that the molecular chaperone, Hsp90 majorly orchestrates stress response signaling and also that compromising Hsp90 function renders cells more responsive to antifungal treatment has been widely established (Cowen 2009). While experimental evidences reinforced the role of Hsf1 not only in the modulation of protein folding in response to heat stress but also under basal conditions (Nicholls et al. 2009). Additionally, apart from its response to thermal stress, Hsf1 is currently associated with additional roles in viability likely due to its role in enabling core gene expression programs, drug response, virulence, filamentation and also iron deprivation mediated response at non-heat shock conditions (Nicholls et al. 2011; Nair et al. 2017). This defines an interconnected transcriptional network at the crossroads between stress responses, cellular integrity, and iron metabolism. Hence, the essentiality of this heat shock factor could be presumed not only to be restricted to the heat shock mediated roles.

Recently, in an attempt to explore the fungicidal effect of a plant alkaloid, berberine, we identified Hsf1 mutant to be most responsive to berberine treatment. Additionally, the mutant also influenced susceptibility of *C. albicans* cells to different drugs with distinct targets (Dhamgaye et al. 2014). While exploring its additional phenotypes under basal conditions, we further established an intricate relationship between cellular iron and Hsf1 mediated drug susceptibility of *C. albicans*. Further, the conditional mutant at room temperature, displayed lower cellular iron pool, leading to enhanced drug susceptibility, dysfunctional mitochondria, and compromised cell wall integrity (Nair et al. 2017). In the present study, we harness chromatin immuno-precipitation coupled to high-density tiling arrays under basal and iron deprived conditions to identify distinct binding sites of Hsf1 in *C. albicans* genome. We identify genes of diverse functions bound by Hsf1 under basal temperatures in both basal and iron deprived conditions. Detailed analysis also led us to identify a novel motif, -GTGn_3_GTGn_3_GTG- where Hsf1 showed strong occupancy at a significant number of sites on several promoters. Additionally, we unravel several iron responsive genes which house Hsf1 binding sites, hence supporting our previous observation that Hsf1 is necessary for cell survival at iron deprivation conditions. The study further highlights Hsf1 role under basal conditions, emphasizing its unconventional roles.

## Materials and methods

### Materials

The growth media YEPD (yeast extract/peptone/dextrose) was purchased from HiMedia (Mumbai, India). BPS (Bathophenanthroline disulfonate), poly l-lysine, tris base, Triton-X 100, NaCl, EDTA and sorbitol were purchased from Sigma chemicals Co. (St. Louis, MO) and ethanol from Merck Millipore. FITC Goat Anti-Rabbit IgG was procured from BD Biosciences, Haryana, India. DAPI (4′,6-diamidino-2-phenylindole, dihydrochloride) was purchased from Life technologies, Delhi, India. Polyclonal anti-Hsf1 antibody used in this study was raised by us previously and checked for specificity (Nair et al. 2017).

### Strains and growth

*Candida albicans* wildtype strain SC5314 *Candida albicans* (ATCC^®^ MYA2876™) was grown in YEPD medium (1% yeast extract, 2% peptone, and 2% dextrose) with and without 150 μM iron chelator BPS in 30 °C incubator at 200 rpm for routine experimental purposes. The wildtype cells under basal conditions without iron deprivation are denoted as BPS (−), while cells treated with BPS are denoted as BPS (+) (or iron deprived) throughout the manuscript.

### ChIP-chip amplification, labeling, hybridization and data analysis

ChIP (Chromatin immuno-precipitation) experiments were performed as previously described (Shukla et al. 2011). In brief, 15 ml of cultures of wildtype [BPS (−)] and iron deprived cells [BPS (+)] were grown till 1 O.D_600_ in YEPD medium. Cells were cross-linked with 1% formaldehyde for 20 min at room temperature. 125 mM glycine was used to quench the cross linking reaction. Cells were then washed twice with ice cold TBS (Tris Buffered Saline: 20 mM Tris–Cl, pH 7.5, containing 150 mM NaCl). After wash, the cells were re-suspended in Zymolyase buffer (50 mM Tris, pH 7.4, 10 mM MgCl_2_, 1 M sorbitol, and 30 mM DTT) containing Zymolyase 20T (Seikagaku Corporation, Japan) at a concentration of 20 μg/ml and incubated for 2 h at 30 °C for the formation of spheroplasts. Spheroplasts were washed twice with cold TBS containing 1 M sorbitol. It was further re-suspended in ChIP lysis buffer (50 mM HEPES–KOH, pH 7.5, 140 mM NaCl, 1 mM EDTA pH 8.0, 1% Triton X-100, 0.1% sodium deoxycholate, and 1 mM PMSF). SDS was added at a final concentration of 0.5% and then the cells were sonicated for 25 s at amplitude of 6 for seven cycles. After sonication, cell suspensions were centrifuged and termed soluble total chromatin (STC). The size of the fragmented chromatin (approximate size ~ 250 to ~ 500 bp) was visualized on 1% agarose gel. A sample of 10% STC, referred to as input DNA, was stored at 20 °C until further use. STC was diluted 5 times with ChIP lysis buffer and pre-cleared with pre-immune serum (negative control). After pre-clearing, anti-Hsf1 antibody was added at a final concentration of 2–5 μg and incubated overnight. The next day, 20 μl packed volume of protein-A agarose beads was added, bound immune complexes were precipitated and washed twice with ChIP lysis buffer, TSE 150 (50 mM Tris–Cl, pH 8.0, 150 mM NaCl, 1% Triton X-100, 0.1% SDS, 1 mM EDTA, pH 8.0, and 1 mM PMSF), TSE 500 (50 mM Tris–Cl, pH 8.0, 500 mM NaCl, 1% Triton X-100, 0.1% SDS, 1 mM EDTA, pH 8.0, and 1 mM PMSF), buffer III (10 mM Tris–Cl, pH 8.0, 1 mM EDTA, pH 8.0, 250 mM LiCl, 1% NP-40, and 1% sodium deoxycholate) and finally with TE (10 mM Tris–Cl, pH 8, and 1 mM EDTA, pH 8.0). The immuno-precipitated complexes were eluted in buffer containing 50 mM Tris–Cl, pH 7.5, 10 mM EDTA, pH 8.0, and 1% SDS at 65 °C overnight. The immuno-precipitated complexes and input DNA were treated with proteinase-K. The input and immuno-precipitated DNA were eluted in 100 and 70 μl TE buffer respectively that were used for ChIP-on-chip experiments.

To study the Hsf1 genome-wide association, ChIPed DNA purified by Qiagen PCR purification kit send to Genotypic technology Bengaluru, for linear amplification by LMPCR (ligation mediated polymerase chain reaction) and size distribution of amplified fragment (~ 250–500 bp) as well as yields were determined. LMPCR was carried out by using 200 ng of input and immuno-precipitated DNA was blunted at 12 °C for 20 min using T4 DNA polymerase as per the Mammalian ChIP-on-chip Protocol Version 10.1 of Agilent Technologies. The blunted DNA was cleaned by phenol:chloroform extraction and the precipitated DNA was re-suspended in 25 μl of water. The entire DNA was ligated to linkers overnight. The linker-ligated DNA was subjected to two rounds of LMPCR and the product was precipitated and re-suspended in 25 μl of water. 2 μg of input, ChIPed or immuno-precipitated DNA were labeled using Agilent Genomic DNA labeling kit PLUS and Cy3 (for ChIPed or immuno-precipitated DNA) or Cy5 (for input DNA) dUTP from Agilent. Using Nano Drop spectrophotometer, the DNA yield and incorporation of label (specific activity) was measured. Labeled DNA were hybridized at 65 °C for 24 h to Agilent 60 mer oligonucleotides. Hybridized arrays were scanned using high throughput Agilent scanner with “SureScan” technology, and process through automated feature extraction using Agilent feature extraction software. The binding profiles were represented as normalized log ratios after quantification and normalization of the signals (ChIPed-DNA or immuno-precipitated DNA/input DNA). Normalization of data and statistical analysis were performed using Agilent’s DNA analytics software (Agilent genomic workbench lite edition 6.5; ChIP-on-chip application, and Microsoft excel). Normalization methods used were median blanks subtraction and intra-array (intensity-dependent) LOWESS normalization. In blanks subtraction normalization for each channel (IP or input) of each replicate of each array, the median of the intensities of the blank spots is subtracted from each intensity on that replicate. As in all normalization steps, any negative signal intensities that result will cause the probe to be flagged as ‘excluded.’ Intra-array normalization attempts to correct for artifacts caused by nonlinear rates of dye incorporation, as well as inconsistencies in the relative fluorescence intensity between some red and green dyes. The LOWESS (locally weighted scatter plot smoothing) algorithm normalizes the channels within each array using a nonlinear polynomial fit to the data, and effectively normalizes by probes and by arrays. Whitehead per-array neighborhood model was used to detect the protein-DNA binding event in DNA Analytics software (DNA analytics detects robust peaks of probe signal corresponding to binding events). This method uses the distribution of all probes on each array to compute robust regions of increased probe signal (termed ‘peaks’). The algorithm does this by examining groups of probe triplets, which are significantly enriched, yielding robust binding event detection. The Whitehead model samples every probe and its immediate upstream or downstream neighboring probe to identify a robust estimate of the location of bound protein (Shariq et al. 2017). Two ChIPed DNA samples were processed for each condition. The data sets that support the results of this article are available in the GEO repository (http://www.ncbi.nlm.nih.gov/geo/) with Accession Number GSE110889 for the tiling array data.

### Immunofluorescence assay

*Candida albicans* strains were freshly revived and inoculated at 0.1 OD_600_ in YEPD. BPS was added at 150 μM concentration in BPS (+) samples. A sample with no BPS was used as control, i.e. BPS (−) samples. Heat shock was induced by rapidly transferring exponential cells to pre-warmed flasks at 45 °C for 30 min. Cells were grown at 30 °C at 200 rpm. For immunofluorescence assay, 1–3 ml of secondary culture (exponential phase cells) was washed 3 times in PBS and fixed in 4% formaldehyde and incubated for 30 min at 30 °C. After fixation, cells were washed twice in PBS (pH 7.5) and re-suspended in 1 ml of 1.2 M sorbitol buffer. 10 μl β-mercaptoethanol and 20 μl zymolyase (5 mg/ml) was then added and cells were incubated at 30 °C with gentle shaking at low rpm (100 rpm) for the formation of spheroplasts. 20 μl cells were adhered to poly-lysine coated cover slips by incubating at room temp for 15 min. Cells were then permeabilized using 0.4% Triton X 100 PBS 7.5 for 5 min. Blocking was done by 1% BSA in PBS. Primary antibody (anti-Hsf1) in PBS + BSA (1:200 ratio) incubation was done at 4 °C overnight. After 3 washes, secondary antibody (FITC Goat anti-Rabbit IgG in 1:500 ratio) and DAPI (4′,6-diamidino-2-phenylindole, dihydrochloride) was added and incubation was done for 2 h at room temperatures. The coverslips were washed and inverted on slides. The imaging was carried out on an inverted Nikon Fluorescence Microscope (Eclipse 90i) at 100×.

### Hsf1 consensus motif discovery

In order to find out the consensus-binding site of Hsf1, the promoter sequences were analyzed with Regulatory sequence analysis tool (RSAT, http://rsat.sb-roscoff.fr) and Multiple EM for Motif Elucidation (MEME) program (http://meme-suite.org/tools/meme). For the analysis of Hsf1 consensus motif, 535 promoter sequences of the genes that showed high Hsf1 binding normalized log ratio and *p* value 0, were selected. 200 bp sequence of the promoter regions were chosen around the detected peaks. The program was allowed to discover motif with oligo and position analysis with oligomer length above 6–7 bp and five motifs per algorithm searched on both strands.

## Results

### Hsf1 occupies core promoter regions both under basal and iron deprived conditions

**Fig. 1:**
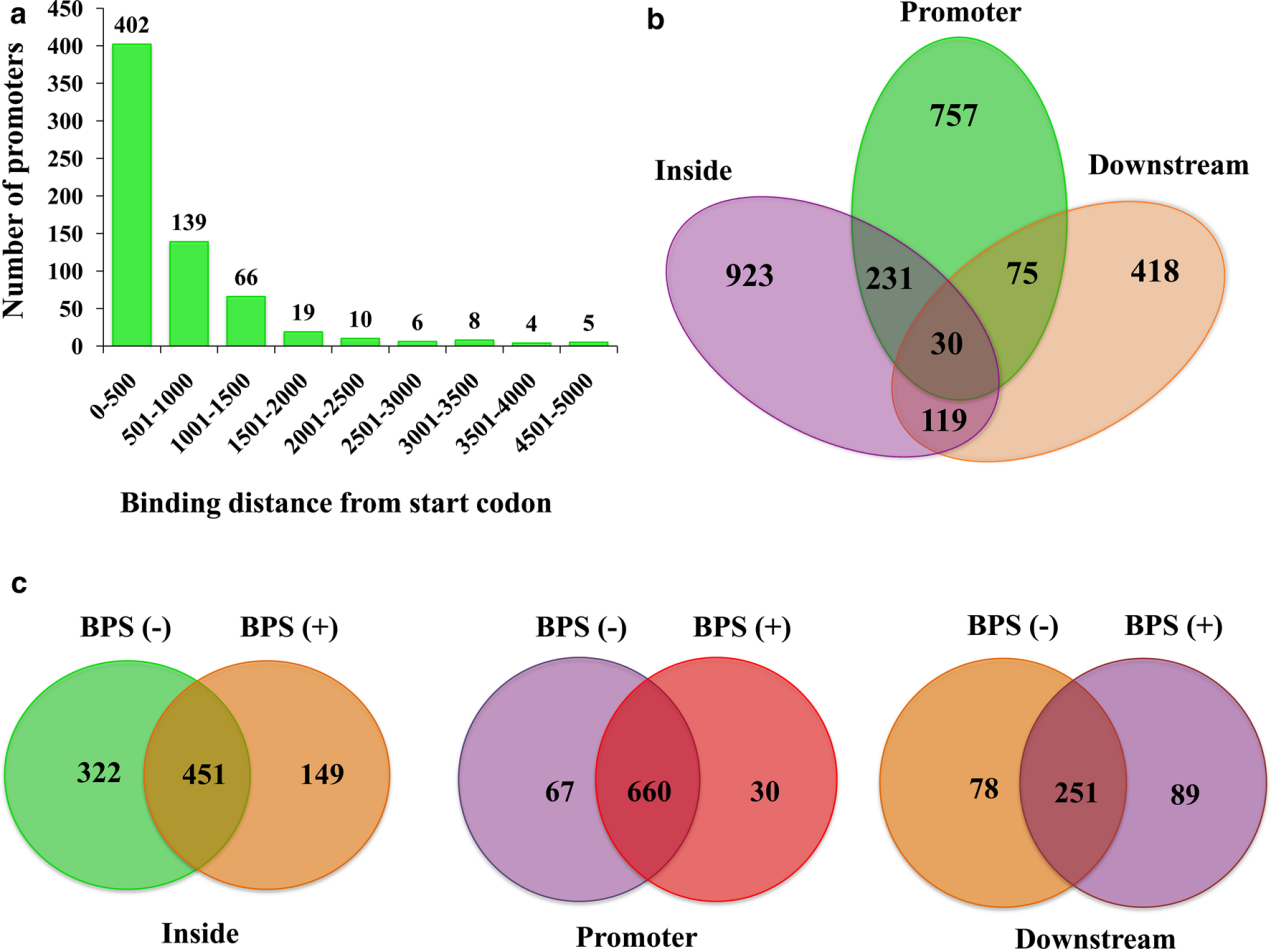
Number of promoters. **a** Binding sites of Hsf1, as determined by ChIP-on chip, were mapped to display their distance from the start codon of the closest genes. Number of promoters for each distance is represented in this histogram. The X-axis represents the binding distance from the start codon and the Y-axis denotes the number of genes. **b** Number of binding sites of Hsf1 seen on the inside, promoter and downstream regions of genes. Common genes are represented in the overlapping regions of the diagram. **c** Comparison of number of binding sites in wildtype and iron deprived cells in inside, promoter and downstream regions

We had shown earlier that the principal coordinator of thermal stress, Hsf1 also presents pleiotropic roles under basal conditions (Nair et al. 2017). In order to get a comprehensible insight into the unconventional roles of this master regulator of heat shock response, chiefly in iron deprivation response, we performed a genome-wide recruitment analysis of Hsf1 using chromatin immuno-precipitation (ChIP)-on chip assay. Our study identified the genome-wide direct targets of Hsf1 in the absence or presence of iron chelator (BPS). Polyclonal anti-Hsf1 antibody was used to pull down the cross-linked protein-DNA complexes. Sample replicates were processed for wildtype [BPS (−)] and iron-deprived cells [BPS (+)] to identify DNA regions that were occupied by Hsf1. Signal peaks detected in the both replicates were chosen. Only promoters with *p* values ≤ 0.05 were considered as significant binding. The average binding profile of the promoter (fragments: − 0/− 500, − 501/− 1000, − 1001/− 1500, − 1501/− 2500, − 2501/− 3000, − 3001/− 3500, − 3501/− 4000 and ≥ 4001, relative to the start codon) showed that the majority of binding events of Hsf1 were found at core promoter regions in both the conditions (Fig. 1a), which however varied in the binding intensities (discussed later).

### *HSF1* binding sites are perceived not only on promoters but also on inside and downstream of genes

Our genome-wide occupancy data analysis revealed that not only does Hsf1 bind to the promoter regions of 757 genes, but also has multiple binding sites on the inside and downstream of the genes (Fig. 1b). In 30 genes including Hsf1 itself, Hsf1 associates strongly in all the three regions, i.e. the promoter, inside and downstream of these genes. Detailed introspection also revealed that Hsf1 occupied 451, 660 and 251 genes that were common under both the conditions on the inside, promoter and downstream regions respectively (Fig. 1c).

Comparative analysis of promoter occupancy revealed that there are 67 gene promoters where Hsf1 showed exclusive occupancy under basal conditions while 30 genes showed exclusive binding under iron deprivation conditions, apart from 660 gene promoters, which were enriched under both the conditions. For all the further data analysis of promoter binding, genes are classified into three main categories (I) Enriched both—660 genes whose promoters are bound by Hsf1 both under basal and iron deprivation conditions. (II) BPS (−) specific (wildtype specific)—67 genes whose promoters are bound by Hsf1 exclusively in basal conditions (III) BPS (+) specific (iron deprived specific)—30 genes whose promoters are bound by Hsf1 specifically on iron deprivation. The binding intensities of the genes of each of these categories are shown in Additional file 1: Table S1.

**Fig. 2:**
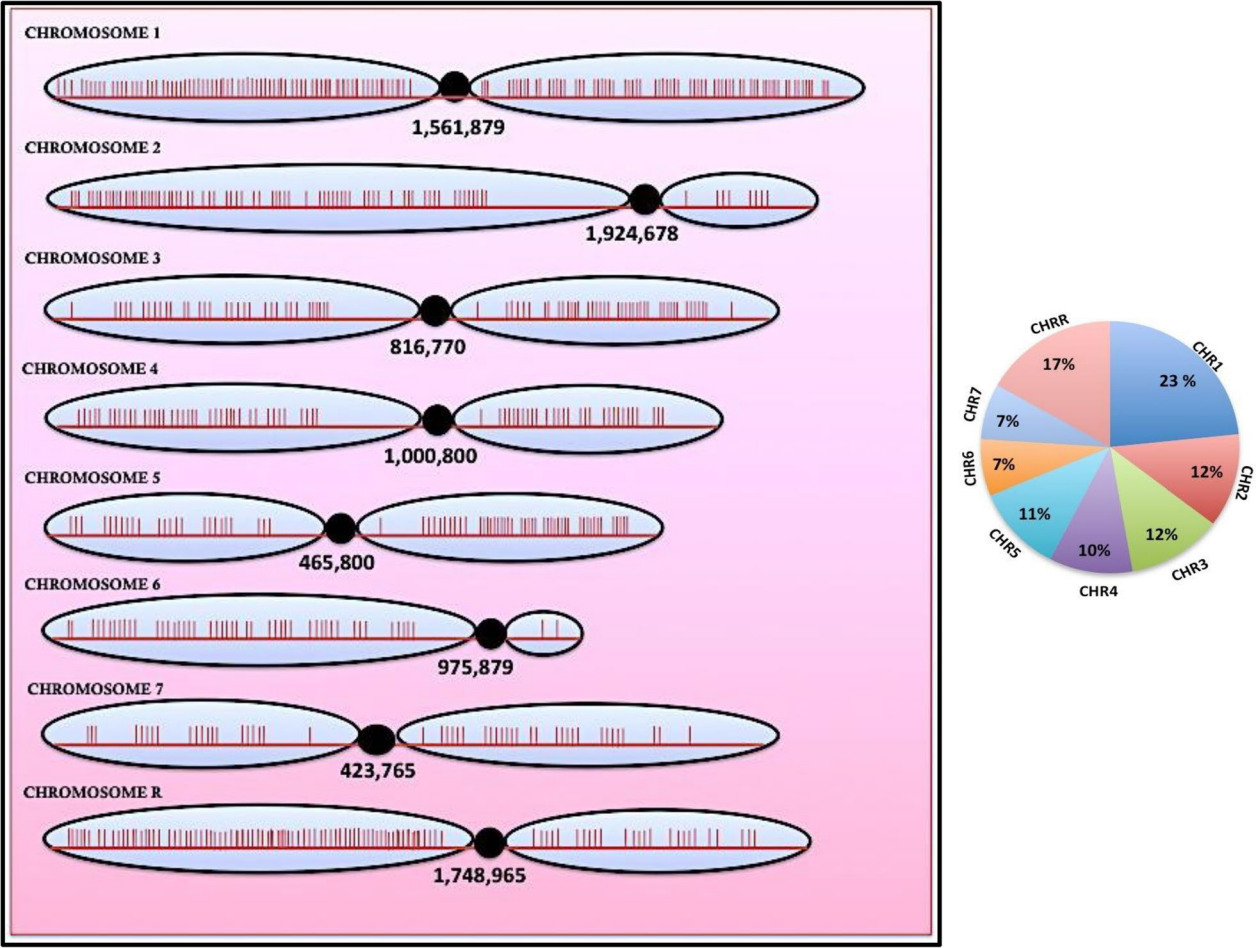
Distribution of Hsf1 binding sites—the distribution of Hsf1 binding sites in the eight Candida albicans chromosomes is represented here. The red vertical bars represent the positions of the Hsf1 binding sites on each chromosome. The black circle indicates the location of the centromere. The pie chart on the right indicates the percentage of Hsf1 binding sites on each chromosome

Further, we inspected the distribution of Hsf1 binding sites on each chromosome for these 660 genes (Additional file 1: Table S2). Notably, chromosome 1 and chromosome R comprised slightly more Hsf1 binding sites 23 and 17% respectively, as compared to the other chromosomes. The Hsf1 binding sites are distributed more or less evenly across the rest of chromosomes varying between 7 and 12% (Fig. 2). In general, the binding sites in each chromosome are primarily located in gene-rich regions and are relatively rare in the centromeres and their flanking regions (Fig. 2). Taken together, these data show that the Hsf1 binding sites are distributed more or less evenly across all chromosomal regions except for the gene-poor centromeres and their surrounding regions.

### Hsf1 occupies genes with diverse metabolic functions

**Fig. 3:**
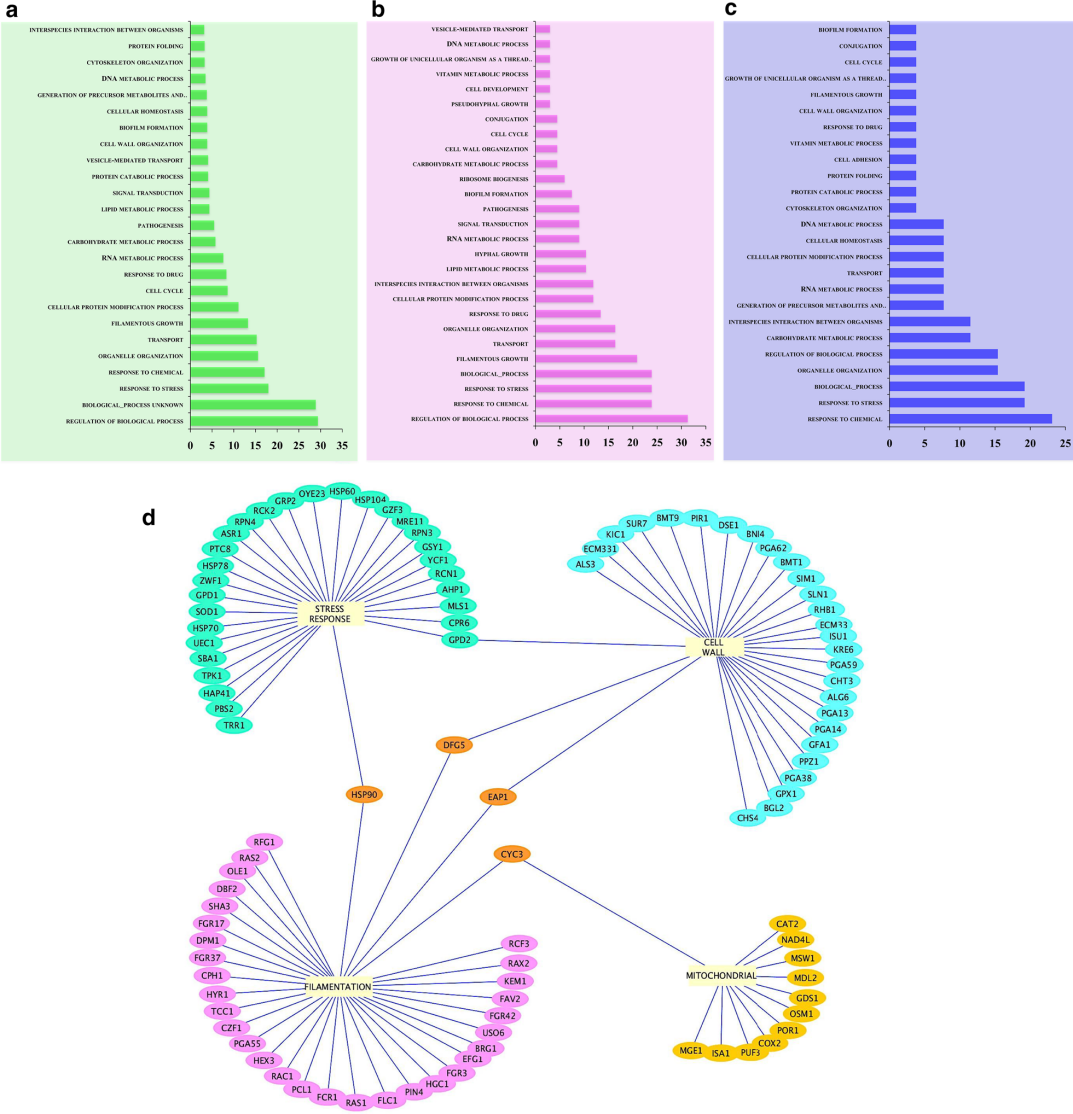
General functional categories of genes enriched by Hsf1—gene ontology (GO) terms of various functions of the genes whose promoters are bound by Hsf1 (**a**) enriched both (**b**) [BPS (−)] specific (**c**) [BPS (−)] specific. Detailed list is provided in Additional file 1: Table S3. The percentage of genes of each category is depicted in Y-axis. X axis represents the functional category. **d** Major functional categories bound by Hsf1—genes of cell wall, mitochondria, filamentation and stress, which show significant promoter occupancy of Hsf1. This network was represented using Cytoscape v3.4.0 software (Seattle, USA)

A detailed analysis of the genes whose promoters were bound by Hsf1 revealed that it binds to genes of diverse functional classes mostly those involved in stress response, response to stimulus, regulation of biological processes, regulation of metabolic functions, filamentation etc. (Fig. 3). Previously, we established that under basal conditions, the heat shock chaperone, Hsf1 played an important role in upholding cell wall and mitochondrial integrity, filamentation and stress response in *C. albicans* (Nair et al. 2017). Closer insights into the binding profile of Hsf1 demonstrates that it enriched the promoters of major genes involved in maintaining cell wall integrity *BMT1*, *BMT9*, *CHS4*, *CHT3*, *ECM33*, *PGA13*, mitochondrial functioning *COX2*, *CYC3*, *MGE1*, filamentation *BRG1*, *EFG1, RAS1*, *RAS2*, oxidative stress *GRP2*, *GSY1*, *SOD1* and osmotic stress *ASR1*, *PBS2*, *RCK2* stress responsive genes. Moreover, Hsf1 also occupied promoters of well-known MDR determinants like *MDR1*, *PDR16*, *ERG11* and *YCF1* (Fig. 3, Additional file 1: Table S3).

Additionally, Hsf1 also elicited constitutive strong binding irrespective of basal or iron deprived conditions on effectors of thermal stress response, i.e. *HSP60*, *HSP 70*, *HSP78*, *HSP90* and *HSP104*. Nicholls et al. had previously demonstrated that Hsf1 governed the significant expression of *HSP90* and *HSP70* even under basal conditions, and their expression was further induced by heat shock (Nicholls et al. 2009). The specific region on these promoters mostly the core promoter regions, where Hsf1 showed maximum enrichment is shown in Additional file 2: Fig S1.

### Hsf1 displayed binding on several iron responsive genes

**Fig. 4:**
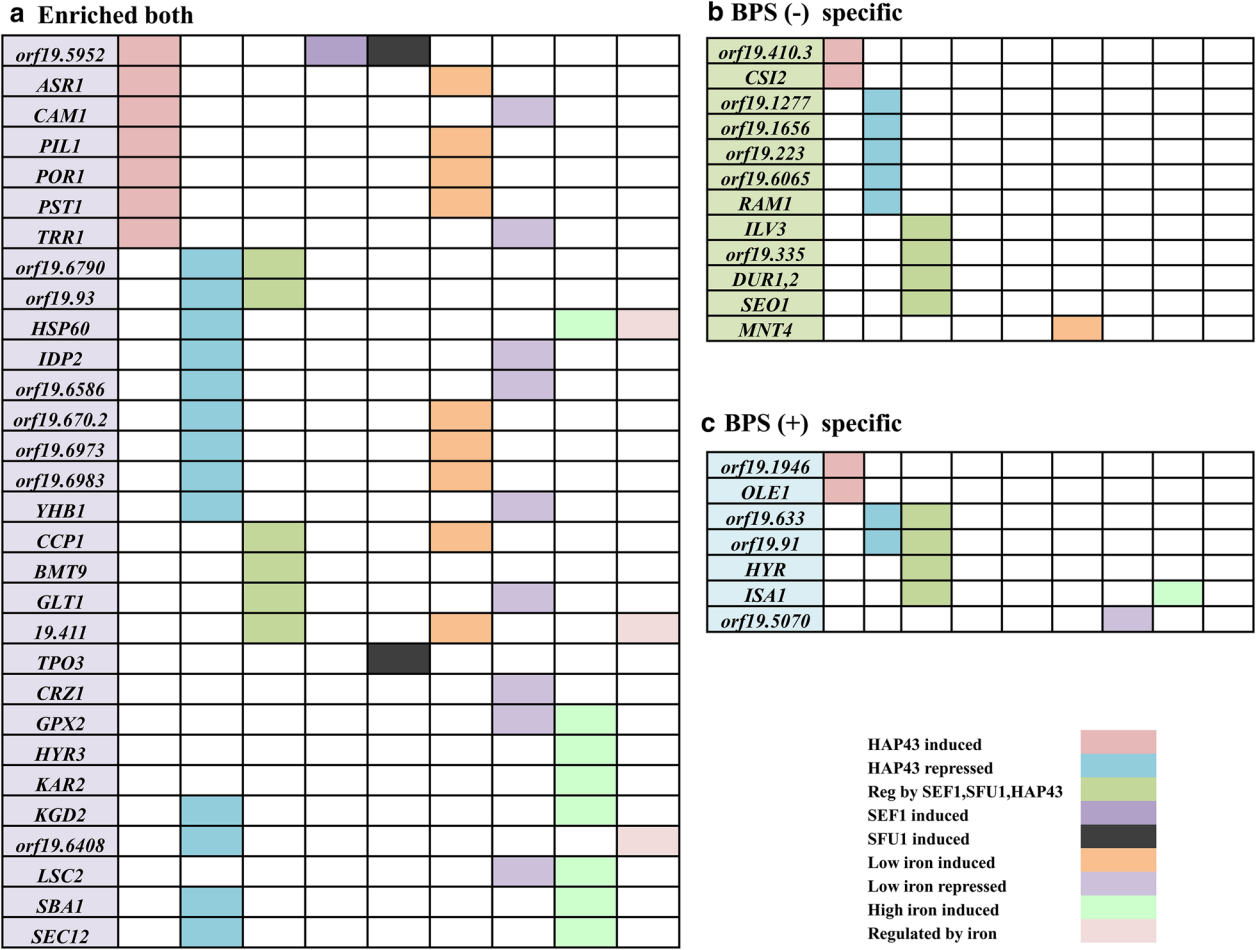
Iron responsive genes elicit Hsf1 binding sites—genes from the three categories [Enriched both (**a**), BPS (−) specific (**b**), BPS (+) specific (**c**)] are examined for either induction or repression by major iron regulators or regulated by iron. They are grouped into various categories and then represented

Earlier, we have observed that intracellular iron levels are tied to Hsf1 levels, which is essential for cell survival under iron-limited conditions. Major transcriptional regulators Sef1, Sfu1 and Hap43, sense and regulate iron deprivation response in *C. albicans* (Chen et al. 2011). A closer look at the promoter-binding pattern of Hsf1 revealed several genes, which are differentially expressed upon iron deprivation also, show Hsf1 binding on their promoters. Also, genes, which are regulated, by Sef1, Sfu1 and Hap43 house Hsf1 binding sites on their promoters. All the three categories wild type exclusive, iron deprived specific and enriched both comprise such genes (Fig. 4). Additionally, we observed that Hsf1 also occupied the promoter regions of Mge1, a mitochondrial chaperone involved in Fe–S metabolism and regulation of susceptibility to drugs, under both basal and iron deprivation conditions (Demuyser et al. 2017).

**Fig. 5:**
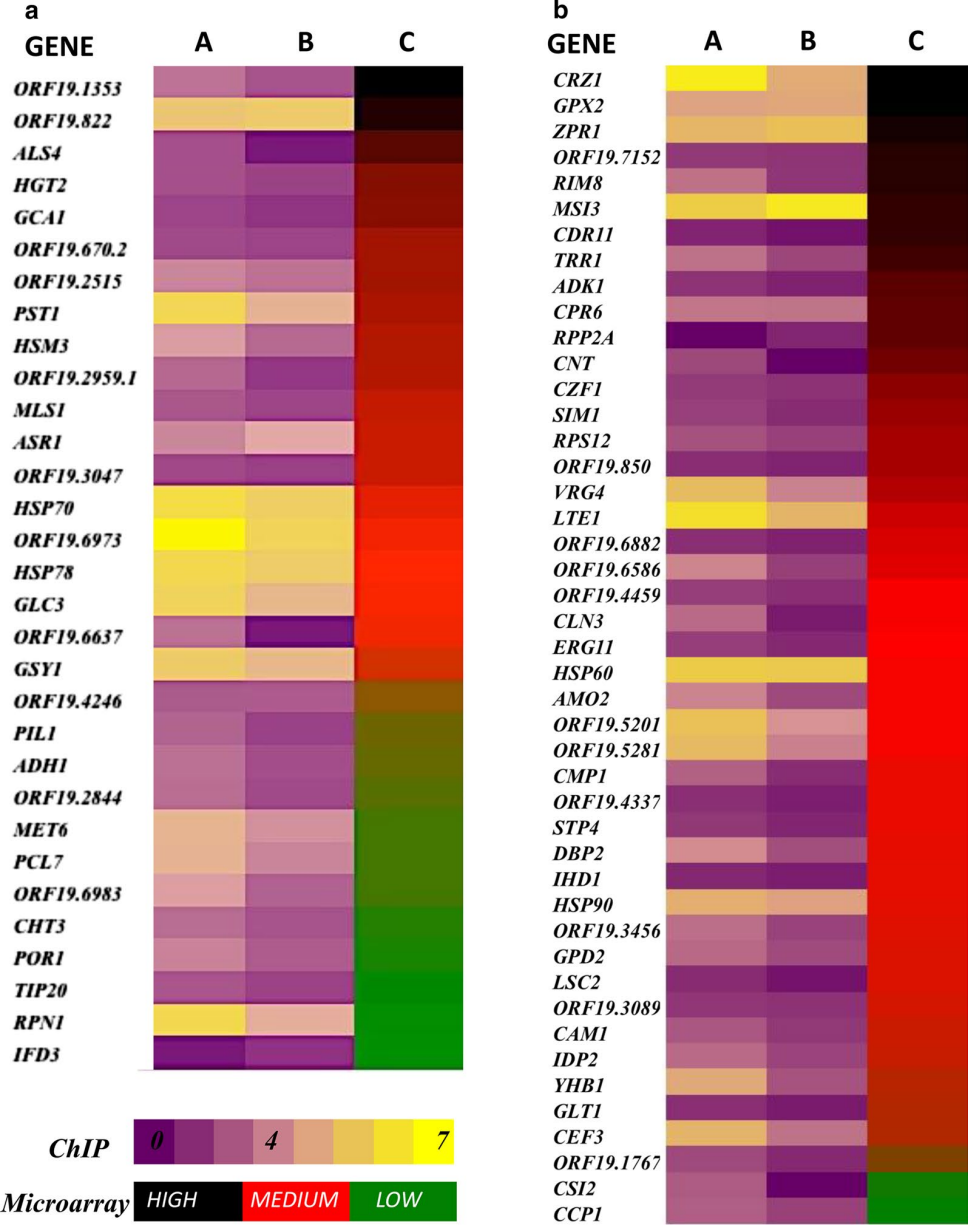
Comparative analysis of microarray and ChIP data under iron deprivation conditions—genes which showed binding of Hsf1 (ChIP data) and also were iron responsive (microarray data) were grouped into two categories. **a** Upregulated upon iron deprivation. **b** Down regulated upon iron deprivation. Column A-binding intensity in wildtype (ChIP) Column B-binding intensity in iron deprivation (ChIP) Column C-mean log fold expression change upon iron deprivation (Microarray). The binding intensities in normal and iron deprived conditions is depicted alongside the fold change in its expression upon iron deprivation according to microarray

The transcript profiling upon iron deprivation has been previously done by us wherein iron deprivation mediated down-regulation of 365 genes and up-regulation of 175 genes in *C. albicans* was observed (Hameed et al. 2011). Here, we carried out a comparative analysis of those iron deprivation responsive genes as seen in microarray analysis and genes whose promoters are bound by Hsf1. Detailed introspection revealed 76 iron responsive genes that show significant Hsf1 binding on their promoters. Out of these, 31 genes are also up regulated in response to iron deprivation while 45 are down regulated (Fig. 5). This provides us a hint about relationship between Hsf1 and iron levels and we hypothesize that binding of Hsf1 to these iron responsive promoters may have a regulatory impact in response to iron deprivation thereby, making Hsf1 essential for survival under iron deprivation conditions.

### Comparative analysis of Hsf1 binding and localization profiles of Hsf1 in wildtype and iron-deprived cells

**Fig. 6:**
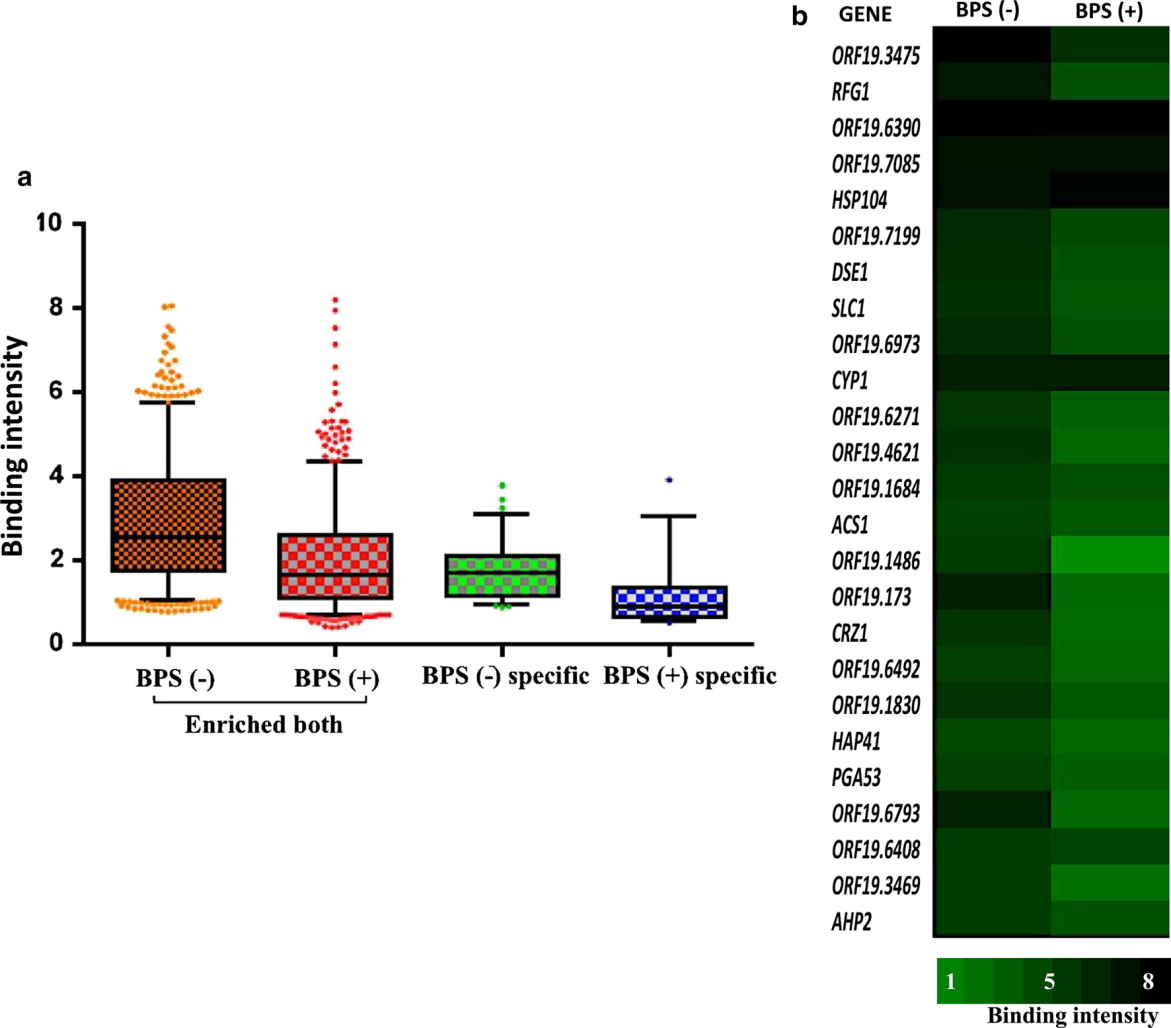
Binding intensities of Hsf1—**a** the distribution of binding intensity are represented by box and whisker plots for all the three categories—enriched both, BPS (−) specific and BPS (+) specific. **b** Top 25 genes which show strongest binding intensities under normal conditions are represented here. Their binding intensities as compared to iron deprived conditions are depicted above

We compared the genome-wide recruitment data of Hsf1 between basal and iron deprived conditions and thereby, identified genes that showed distinct occupancy of Hsf1 at the promoter regions. The analysis revealed that the binding profile of Hsf1 under various categories [enriched both, BPS (−) specific, BPS (+) specific] differed in the intensity of significant binding in terms of *p* values (as depicted by normalized log ratios) and the range varied from a highest log ratio of 8.4 to a lowest of 1 (Fig. 6a). Further analysis revealed that a higher range of binding intensity was observed under basal conditions as compared to iron deprived conditions as seen in Fig. 6a. The top 25 genes displaying strongest binding intensity of Hsf1 on gene promoters are depicted in Fig. 6b.

**Fig. 7:**
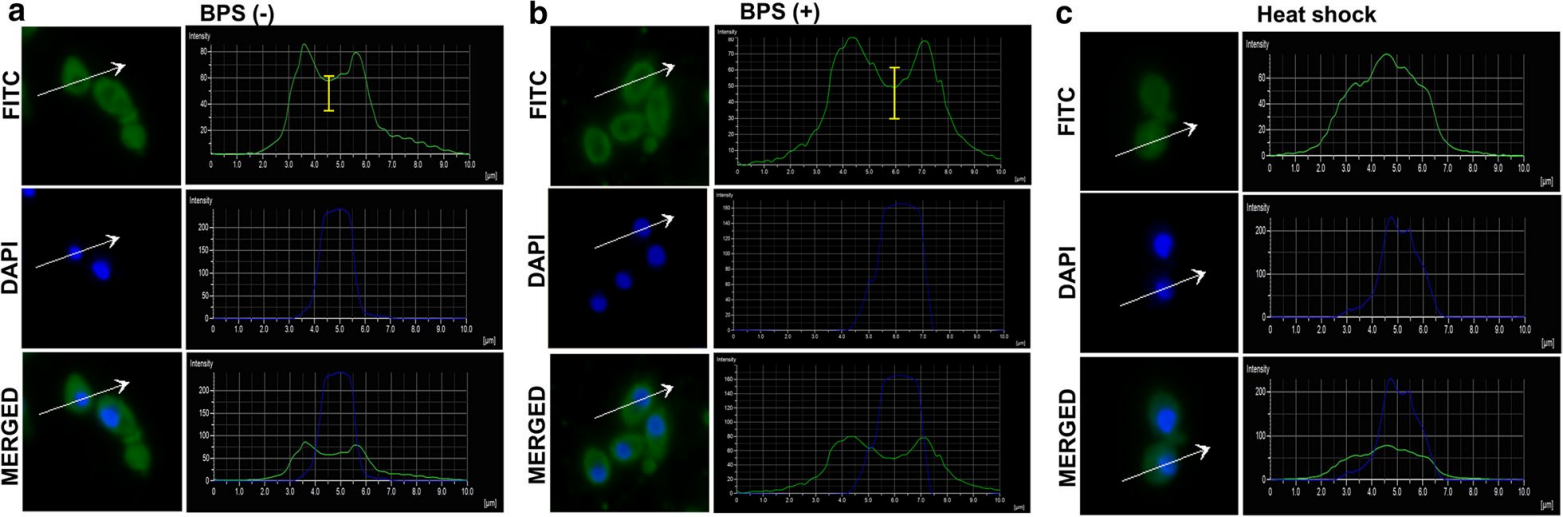
Analysis of cellular localization of Hsf1—Microscopy images of Hsf1 cellular localization in **a** wildtype [BPS (−)] cells, **b** iron deprived [BPS (+)] cells and **c** heat shock treated cells. The fluorescent images are shown alongside quantified intensity plot. The topmost panel in each section represents staining with primary antibody (anti-Hsf1 coupled with secondary antibody labeled with FITC visualized as green fluorescence). The middle panel represents nuclear staining with DAPI. The bottom panel is the merged Fig. The fluorescence intensity of the selected transversal section of the cell (arrow line) was quantified and represented as intensity plot, where the length and direction are expressed as distance on the x-axis. Two peaks in the intensity plot in **a** and **b** corresponding to cytoplasmic localization of Hsf1 was observed. The dip in fluorescence depicting Hsf1 concentration in the nucleus in **a** and **b** panel is due to the lesser amount of Hsf1 in the nucleus as compared to the cytoplasm. The dip is more prominent in case of BPS (+) cells. The sharp peak in case of heat shock **c** corresponds to increased nuclear localization of Hsf1 upon heat shock conditions

Previously it has been established that Hsf1 translocates from the cytoplasm to the nucleus in response to heat stress in order to induce the expression of required proteins to combat heat stress (Nakai and Ishikawa 2000). Here, we tested whether iron deprivation could impact the translocation of Hsf1, which in turn could be reflected in presently observed differential binding intensities in basal and iron deprivation conditions. For this, we employed polyclonal Hsf1 antibody and compared its localization in basal and iron deprived conditions by immunofluorescence as described in “Materials and methods”. *C. albicans* cells were subjected to 30 min heat shock and were used as a positive control since it is established from previous studies that Hsf1 gets preferentially localized into the nucleus upon thermal stress. Under basal conditions immunofluorescence images exhibited that Hsf1 was mostly dispersed throughout the cytoplasm in wildtype and iron deprived cells with certain levels localizing into the nucleus as well. Interestingly, quantification of fluorescence in iron-deprived cells across the cells as compared to the wildtype showed that cells treated with BPS displayed comparatively lower fluorescent intensities in the nucleus (approx 12% lower), which is depicted as a more prominent depression in the intensity plot in BPS treated cells (Fig. 7a, b). Expectedly, in the present study cells subjected to heat shock showed a peak in the nucleus regions signifying Hsf1 nuclear localization upon thermal stress (Fig. 7c).

**Fig. 8:**
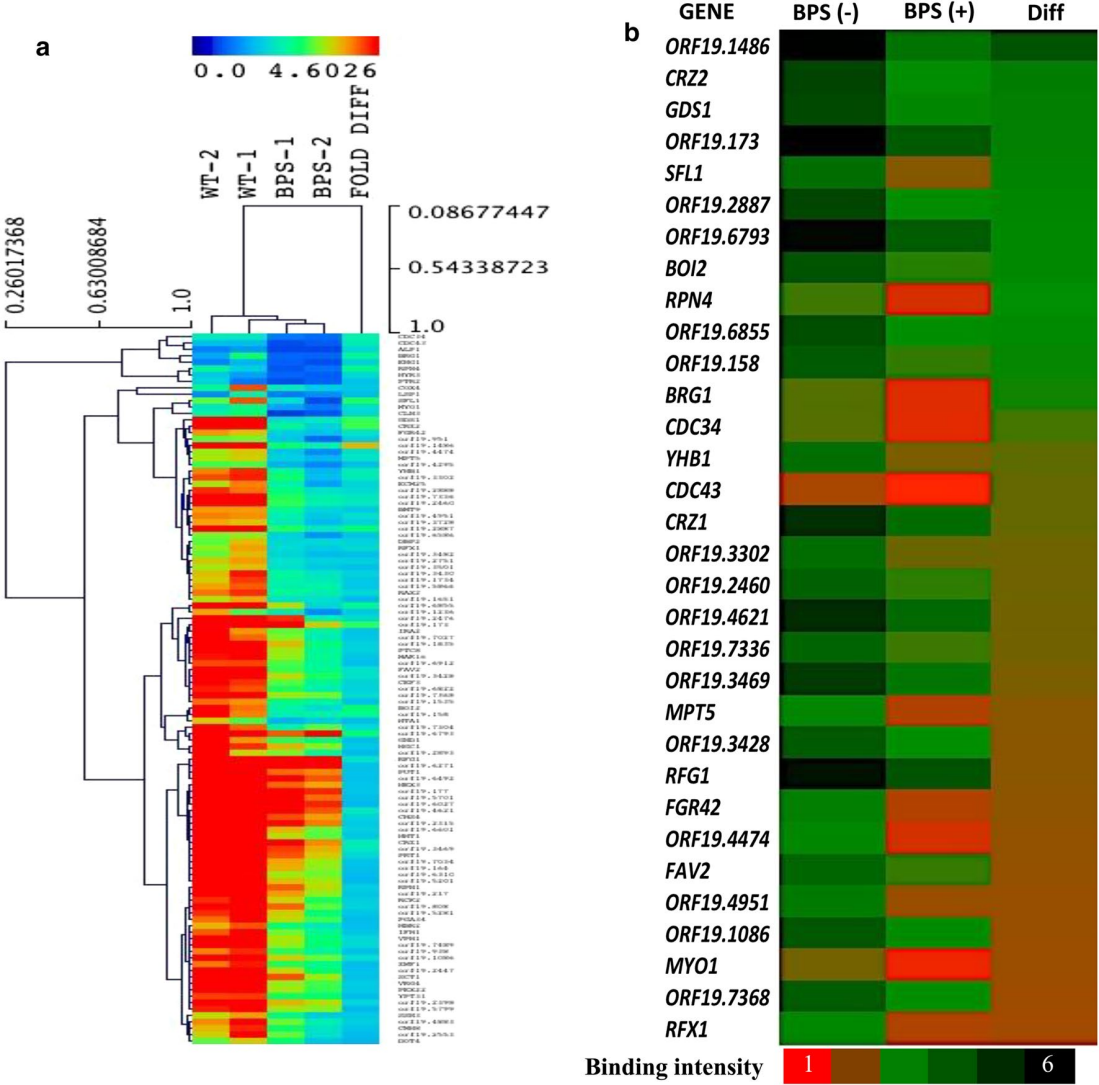
Comparison of Hsf1 binding intensity under wild-type and iron-deprived conditions. **a** Differences of binding intensity of genes whose promoters are enriched both under normal and iron deprived conditions using Jalview Software. **b** The genes which show maximum difference in intensities are represented as heat map

Additionally, comprehensive analysis of the genome wide data, specifically those genes, where Hsf1 shows significant binding both under basal and iron deprived conditions (enriched both category) was carried out. These genes were analyzed and sorted on the basis of difference in binding intensity in basal conditions as compared to iron deprivation (Fig. 8a). Interestingly, on a detailed analysis, it was observed that Hsf1 showed approximately 3.7 times lower binding intensity on the promoter of an uncharacterized gene, *orf 19.1486*, under iron deprivation as compared to binding under wildtype conditions (Fig. 8b). In silico analysis revealed that this protein is localized to the nucleus. All other genes for example, the calcineurin pathway genes *CRZ1*, *CRZ2*, major regulator of nitrosative stress, *YHB1*, and a few other genes also displayed high differences (approx > 2), however lesser than the binding intensity 3 as seen in the case of *orf 19.1486*.

### Novel predicted motifs of Hsf1 under basal conditions in *Candida albicans*

The underpinnings that not only does Hsf1 bind to the canonical HSE [(nGAAn)_3_] constitutively, but also to some non canonical binding sites (GAAnnTTC and TTCn_7_TTC) upon heat shock induction in order to regulate the expression of *C. albicans* genes has been recently reported (Leach et al. 2016). We examined whether Hsf1 performs its diverse functions under basal conditions by binding to motifs similar to heat shock elements (HSEs) or employ other independent motifs. For this a de novo motif discovery by employing MEME software was carried out. Out of the 660 genes which showed Hsf1 enrichment on promoters under both wildtype and iron deficient conditions, we selected 535 genes with the p-value = 0 (denoting most significant enrichment of Hsf1) and then analyzed along with 535 control sequences for motif analysis. 200 bp spanning the summit of Hsf1 peaks was extracted for all the 535 sequences for motif discovery.

**Fig. 9:**
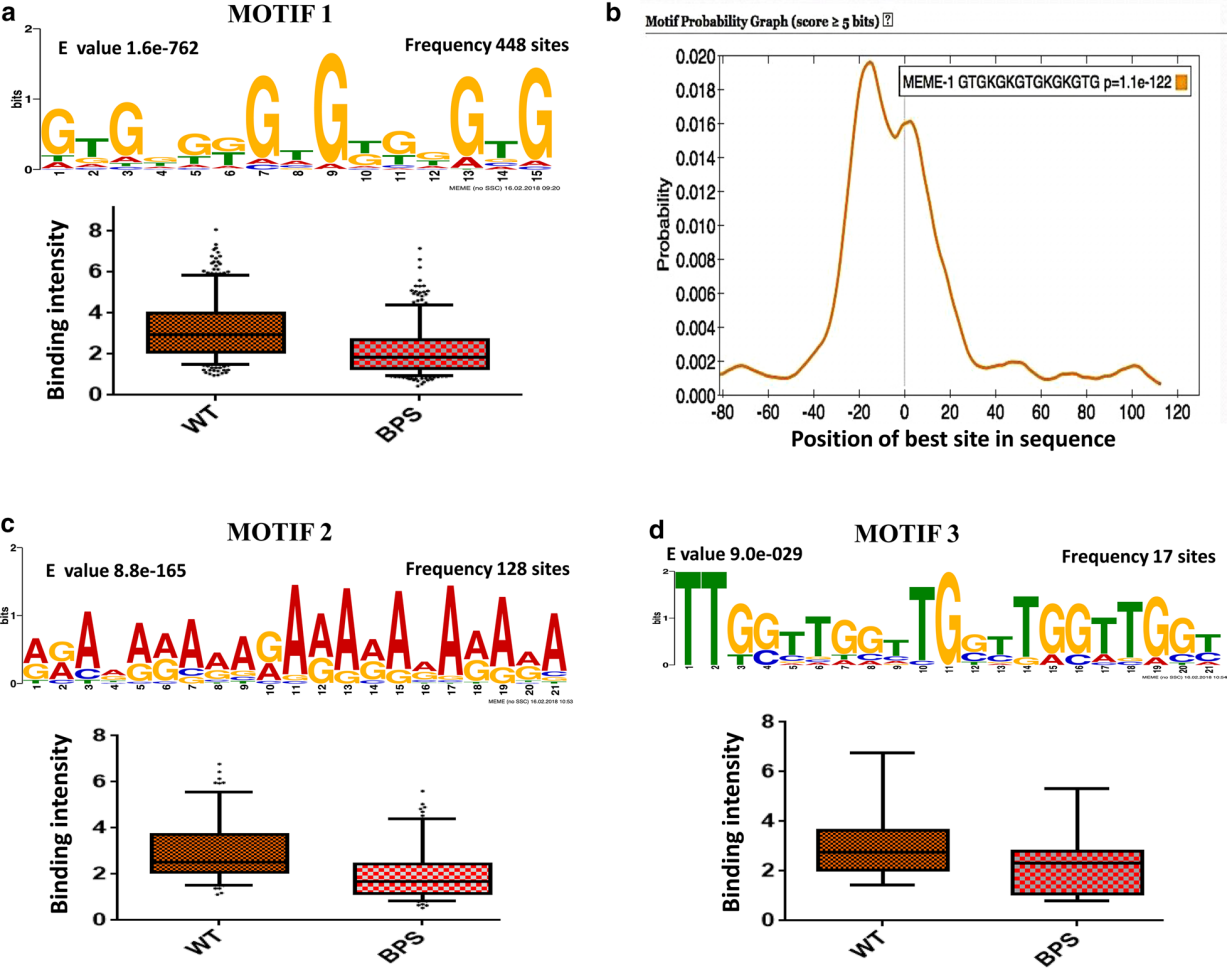
Hsf1 binding sites prediction: Hsf1 binding peaks are predicted based upon ChIP, peaks using MEME software. (**a**) Motif 1-De novo motif analysis using MEME on Hsf1-binding sites identified this highly significant sequence (E-value = 1.6 e–762). This motif is centrally enriched as seen in plot (**b**). The motifs with second and third highest frequency are represented as motif 2 (**c**) and motif 3 (**d**)

The presence of multiple binding motifs of a transcription factor most commonly is associated with their mobility especially in the case of stress responsive transcription factors, so that they can activate myriad of genes upon encountering stress condition. Interestingly, analysis revealed a novel motif—(GTGn_3_GTGn_3_GTG) (Fig. 9a), which was centrally enriched (Fig. 9b) and also showed the highest frequency of occurrence, 448 sites in total as compared to the other motifs identified along with the canonical [(nGAAn)_3_] motif. Two other motifs also showed significant enrichment, dispersed at different locations among the 535 genes (Fig. 9c, d) although at a lower frequency. Further investigations showed that, Hsf1 displayed differential binding affinities under basal and iron deprived conditions in all the three different Hsf1 motifs discovered.

## Discussion

In the commensal human pathogen, *C. albicans,* Hsf1 is recently reported to be essential for survival under iron deprivation conditions wherein; its conditional mutant elicited lower intracellular levels of iron and consequently dysfunctional mitochondria (Nair et al. 2017). In an attempt to understand the regulation of non-conventional roles mediated by Hsf1, and thereby, deciphering its transcriptional network especially in the context of iron homeostasis, we employed genome-wide chromatin immuno-precipitation studies under basal and iron deficit conditions in *C. albicans* cells. A deeper insight into the ChIP data revealed that the transcriptional regulation by Hsf1 is not only restricted to the induction of heat shock proteins (Hsps) but also to several genes across the entire genome which displayed its moderate to high occupancy on its promoter. The Hsf1 binding sites spanned not only to core gene promoters but also to the downstream and inside regions. It is usually noted that binding just upstream of the transcription start site (TSS) helps in positioning the polymerase II at the beginning of the gene for transcriptional activation. Presence of transcription factor (TF) binding sites inside or downstream of gene may either act as regulatory sequence for the same gene or as an enhancer for the control of same gene or downstream gene (acting as a distal enhancer) in orientation and position independent manner. However, all the binding sites may not be functional or represents a non-productive binding site or could just be alternative TSS (Shariq et al. 2017).

Genomic analysis uncovered that Hsf1 binds to genes of pleiotropic functionality mainly those implemented in stress response, cell wall integrity, filamentation, nutrient stress response and several mitochondrial genes, thereby, highlighting the global role of this heat shock transcription factor under basal conditions. The fact that Hsf1 levels affected susceptibility to various cell wall perturbing agents and that the cell wall integrity in Hsf1 is also compromised, matched well with present observation that several major regulators of cell wall *RHB1*, *SIM1*, S*UR7*, etc. involved in cell wall maintenance apart from *CHT3* and *CHS4* the principal components in chitin biosynthesis show strong promoter occupancy of Hsf1 (Dünkler et al. 2005). A few prime coordinators of filamentation comprising *RAS1, RAS2* and *EFG1* also have the occupancy of Hsf1 on its promoters, strengthening our previous observation that Hsf1 regulates morphogenesis under basal conditions (Feng et al. 1999; Desai et al. 2015). Additionally, the influence of Hsf1 on mitochondrial integrity was evident not only from the inability of the Hsf1 mutant to grow on non-fermentable carbon sources but also failure to take up Mitotracker red stain. Here, we observed that Hsf1 displayed significant enrichment on the promoters of cytochrome c subunits, *COX2* and *COX4* oxidase, a mitochondrial matrix co-chaperone, *MGE1* also seen to have a role in iron homeostasis, apart from several other mitochondrial genes (Demuyser et al. 2017). Our previous observation depicting the essentiality of Hsf1 for growth in iron-deprived media could be attributed to the fact that various iron responsive genes which are mostly regulated by the major iron regulators Sef1, Sfu1 and Hap43 are also regulated by Hsf1 as visualized by strong binding on their promoters (Fig. 4). In this context, it is relevant to mention that certain heat shock chaperones have been intimately linked for their roles in iron homeostasis previously. Generally, the Fe–S cluster chaperone system dedicatedly mediates the process of cluster release from the scaffold for transfer to the target proteins to cytoplasm, nucleus or mitochondria. Attenuation of any component of the yeast Fe–S cluster assembly and export systems not only results in Fe/S protein defects, but also has severe consequences for intracellular iron homeostasis (Lill et al. 2006). Recent evidences show instances of certain chaperones specially of the Hsp70 family of proteins are involved in Fe–S cluster delivery and this interactions are not only restricted to yeast but are also observed in humans wherein, the chaperones comprise heat shock protein HSPA9 and the J-type chaperone Hsc20 (Wachnowsky et al. 2018). Hence, suggesting wider roles of these heat shock factors and their iron-mediated effects.

The most noteworthy binding of Hsf1 was on uncharacterized *orf 19.1486,* which showed maximum differential binding intensity under iron-deprived conditions. Earlier, the protein codded by *orf 19.1486* has been shown to possess a life-span regulatory factor domain regulated by Sef1, Sfu1, and Hap43. Additionally, in cells lacking the iron regulator Sef1, the expression of *orf 19.1486* is reported to be down regulated (Chen et al. 2011). Hence, this *orf* appears to be a common target of both the major regulator of iron homeostasis, Sef1 and also the thermal stress regulator Hsf1, however, this remains to be explored.

The transcriptional landscape of *C. albicans* cells had revealed that greater number of genes were down regulated upon iron scarcity, while, the heat sock proteins *HSP30*, *HSP70*, *HSP78, HSP60* and *HSP90* manifested variable transcript levels upon iron deprivation (Hameed et al. 2011). In this study, we observed that Hsf1 display strong and significant binding on these gene promoters both under basal and iron deprived conditions, interestingly, with similar binding intensities. This may be indicative of the fact that the association of Hsf1 on these genes is essential irrespective of the variable environmental cues. Notably, under basal conditions, we observed majority of the genes to have a stronger binding intensity of Hsf1 in basal conditions as compared to iron deprived situation. This could be linked to decrease translocation of Hsf1 to the nucleus upon iron deprivation as suggested by our immunofluorescence assays (Fig. 7). How iron deficiency impacts Hsf1 intracellular translocation need to be explored. One could speculate that iron may be essential for proper functioning or targeting of Hsf1 to the nucleus.

Owing to their similar DNA binding domains, eukaryotic transcription factors (TFs), have been grouped into various families. However, there may be multiple binding sites for certain TFs in order to enable distinct gene expression programs depending on the environmental cues. For instance, recently the transcription factors from ZCF family displayed binding on multiple motifs in response to oxidative stress conditions (Issi et al. 2017). Our analysis revealed a new additional motif -GTGn_3_GTGn_3_GTG-, where Hsf1 showed significant enrichment in several gene promoters under basal conditions. Notably, this is distinct from the well-known -nGAAn- repeat motif enriched mostly in response to heat shock and therefore, called as Heat Shock Element (HSE) (Nicholls et al. 2011). The presence of alternate motifs or rather the absence of a dominant binding motif likely facilitates the mobility of such stress responsive transcription factors involved in varied functions. Interestingly, the enrichment of Hsf1 on these motifs also varied in intensities depending on presence or absence of iron.

While the precise nature of the signal eliciting the heat shock response remains explicit in humans and yeast, it is the non-heat shock roles of Hsf1 are beginning to be realized. Additionally, in a parallel study carried out in model yeast, it was observed that Hsf1 performs essential function in yeast under all conditions while mammalian Hsf1 is dispensable for normal cell growth but becomes essential during oncogenic transformation (Solís et al. 2016). Present study highlights the non-heat shock responsive roles of Hsf1. The involvement of heat shock proteins (Hsps) upon nutrient deprivation or alternate stresses may act as an immediate line of defense in providing a cross protection against several direct or indirect stresses. Together, our data sheds light on the genes and biological functions controlled by Hsf1 which plays a critical role in drug resistance, iron deprivation, filamentation and general stress response in *C. albicans*. Hsf1 hence, also orchestrates diverse transcriptional programs in *C. albicans* under basal conditions.

## Additional files

**Additional file 1.** Additional tables.

**Additional file 2.** Additional figure.
