# Supplementary material for: Identification of genome-wide binding sites of heat shock factor 1, Hsf1, under basal conditions in the human pathogenic yeast, Candida albicans
Source: AMB Express. 2018 Jul 16;8:116. doi: 10.1186/s13568-018-0647-7 (PMC6047955; doi:10.1186/s13568-018-0647-7)
Supplement: Supplementary file 2 — Additional file 2. Additional figure. [file 13568_2018_647_MOESM2_ESM.pdf]

Journal name	**AMB Express**
Manuscript Title	**Identification of genome-wide binding sites of Heat shock factor 1, Hsf1, at basal conditions in the human pathogenic yeast, *Candida albicans***
The name(s) of the author(s)	Remya Nair[1,2], Nitesh K. Khandelwal[2,3],Md.Shariq,[2,4], Archana K. Redhu[2,5], Naseem A. Gaur[3], Shamim Shaikh[1] and Rajendra Prasad[6*]
The affiliation(s) and address(es) of the author(s)	[1] Rajiv Gandhi Institute of IT & Biotechnology, Bharati Vidyapeeth University, Pune
	[2]School of Life Sciences, Jawaharlal Nehru University, New Delhi,110067, India.
	[3] International Centre for Genetic Engineering and Biotechnology , New Delhi,110067, India.
	[4]National Institute of Pathology, Safdarjung campus, New Delhi, 110029, India.
	[5]Department of Biosciences and Bioengineering, IIT-Bombay
	[6]Amity Institute of Integrative Sciences and Health and Amity Institute of Biotechnology Amity University, Gurgaon, 122413, India
The e-mail address of the corresponding author	**rprasad@ggn.amity.edu , rp47jnu@gmail.com**
**Figure Legend**	
**Supplementary Figure 1-**	**Figure representing the specific region on the promoters of *HSP60, HSP70, HSP78, HSP90* and *HSP104* where maximum intensity of binding is observed. The value of comparative binding intensities between wildtype and iron deprived is represented as green and black colour. Black colour represents stronger binding.**

Figure S1

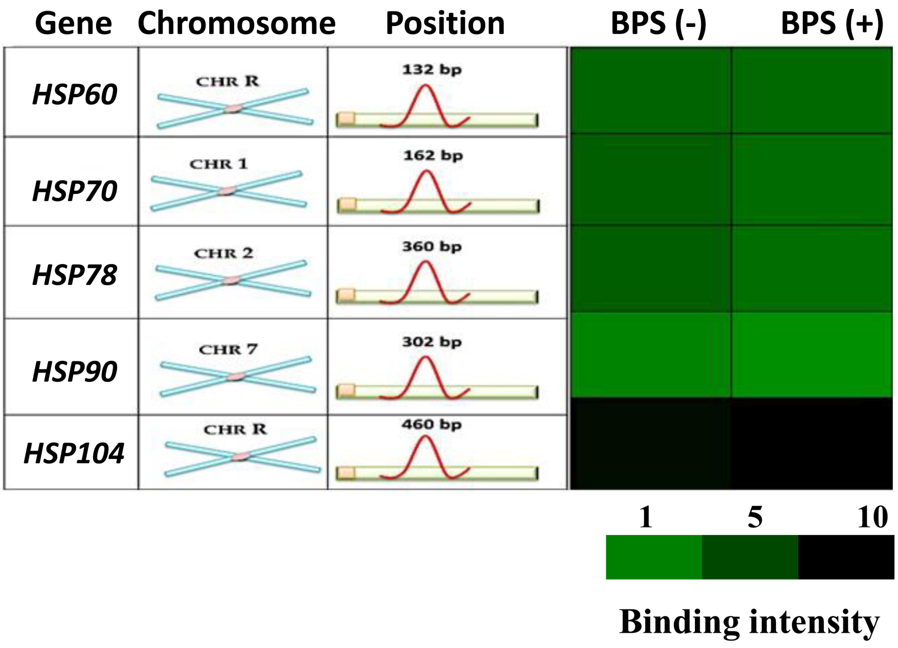
Gene
Chromosome
Position
BPS (-)
BPS (+)
HSP60
CHR R
132 bp
HSP70
CHR 1
162 bp
HSP78
CHR 2
360 bp
HSP90
CHR 7
302 bp
HSP104
CHR R
460 bp
1
5
10
Binding intensity
